# Treatment cure rate and its predictors among children with severe acute malnutrition in northwest Ethiopia: A retrospective record review

**DOI:** 10.1371/journal.pone.0211628

**Published:** 2019-02-20

**Authors:** Fasil Wagnew, Getiye Dejenu, Setegn Eshetie, Animut Alebel, Wubet Worku, Amanuel Alemu Abajobir

**Affiliations:** 1 College of Health Sciences, Debre Markos University, Debre Markos, Ethiopia; 2 College of Health Sciences, University of Gondar, Gondar, Ethiopia; 3 Faculty of Medicine, The University of Queensland, Brisbane, Australia; Sefako Makgatho Health Sciences University, SOUTH AFRICA

## Abstract

**Background:**

More than 29 million that is an estimated 5%, under-five children suffer from severe acute malnutrition (SAM) globally, with a nine times higher risk of mortality than that of well-nourished children. However, little is known regarding outcomes and predictors of SAM in Ethiopia. Therefore, this study aims to determine treatment cure rate and its predictors among children aged 6–59 months with SAM admitted to a stabilization center.

**Methodology:**

A retrospective record review was employed in SAM children at the University of Gondar Comprehensive Specialized Hospital (UOGCSH) from 2014 to 2016. SAM defined as weight for height below -3 z scores of the median World Health Organization (WHO) growth standards or presence of bilateral edema or mid upper arm circumference < 115mm for a child ≥6months age. All SAM patients with medical complication(s) or failure to pass appetite test are admitted to the malnutrition treatment center for inpatient follow-up. Data were extracted from a randomly selected records after getting ethical clearance. Data were cleaned, coded and entered to Epi-info version-7, and analyzed using STATA/se version-14. Descriptive statistics and analytic analyses schemes including bivariable and multivariable Cox proportional hazards model were conducted.

**Result:**

Among a total of 416 records recruited for this study, 288 (69.2%) SAM children were cured at the end of the follow up, with a median cure time of 11 days. Kwash-dermatosis (AHR (Adjusted Hazard Ratio): 1.48(95% CI: 1.01, 2.16)), anemia (AHR: 1.36(95% CI: 1.07, 1.74)), tuberculosis (AHR: 1.6(95% CI: 1.04, 2.43)) and altered body temperature at admission (AHR: 1.58(95% CI: 1.04, 2.4) were independent predictors of time to cure.

**Conclusion:**

The cure rate in SAM children was low relative to sphere standard guideline. Prognosis of SAM largely depends on the presence of other comorbidities at admission. Available intervention modalities need to address coexisting morbidities to achieve better outcomes in SAM children.

## Background

Childhood under-nutrition refers to a combination of nutritional disorders that include underweight (mixed), wasting (acute), stunting (chronic) and micronutrient deficiency (2). Wasting (weight for height) is an acute malnutrition due to a recent failure to receive adequate nutrition and may be affected by recent episodes of diarrhea and other acute illnesses (3). Based on severity, acute malnutrition is classified as moderate acute malnutrition (MAM) and Severe Acute Malnutrition (SAM) (5).

More than 29 million, that is an estimated 5%, suffer from SAM globally, with a nine times higher risk of mortality than that of well-nourished children [1, 2]. Indeed, SAM in children is a major public health problem in developing nations [3] including sub-Saharan Africa[4]. For instance, 42.3% children are acutely malnourished, with 16.3% severely wasted in Dollo Ado district of Somalia region (Ethiopia) [5]. According to Health and Health Related Indicators (HHRI) 2014, in Ethiopia; SAM was the third leading cause of mortality accounting for 8.1% of under-five children deaths [6]. This might be due to immune-compromization from complex, adaptive physiologic and metabolic processes secondary to insufficient nutrients [7]. Underlying and/or concurrent medical conditions including dehydration, anemia, sepsis, hypoglycemia and hypothermia [8] might also contributes to this high death toll.

Prognosis for SAM treatment continues to be a challenge [9] and better outcomes for inpatient interventions still remains low due to co-morbidity [10–14], poor adherence to treatment guideline, mismanagement of cases and other socio-demographic factors [10, 15–19]. As a result, health sector has upgraded nutritional interventions through the health promotion, effective treatment strategy and supplementation of essential micronutrients for children and mothers [3, 20, 21]. Nearly, 303,000 children under the age of five are at risk of SAM and 130,000 children required treatment for SAM in 2017 alone. Most Ethiopian regions including parts of Southern Nations, Nationalities, Amhara and Oromia have malnutrition problems due to seasonal variations that might lead to poor crop production [22] and other factors at multiple levels. However, little is known about cure rate of SAM treatment and its predictors in Ethiopia. This study aims to determine treatment cure rate and its predictors among children aged 6–59 months with SAM admitted to a stabilization center in UOGCSH.

## Methods

### Study area and design

An institution-based, retrospective record review was employed at UOGCSH. The hospital serves as a referral center for North Gondar administrative district and residents from catchment areas. It has 512 beds, of which 70 beds are allocated to pediatrics ward. This ward has a separate room (center) for treatment of malnourished children. Health personnel follow an updated and standardized treatment SAM management guideline [23]. Based on this guideline, all SAM patients with medical complication(s) or failure to pass appetite test are admitted to the malnutrition treatment center for inpatient follow-up and effective treatment.

### Population

The source population were all records children aged 6–59 months with SAM admitted to Therapeutic Feeding Center (TFC) at the UOGCSH from January1/2014 to December 30/ 2016. The study randomly selected eligible children with SAM admitted to a stabilization center at the hospital from 1 January 2014 to 30 December 2016. A total of 1,027 children with SAM were admitted to the hospital from January1/2014 to December 30/ 2016. All children 6–59 months of age with SAM that have been admitted and treated at inpatient TFU of the hospital from January1/2014 to December 30/2016 were included in the study. However, children with incomplete records with regard to variables of interest such as baseline sociodemographic characteristics and patient treatment outcomes (i.e. cure, death, and defaulter) were excluded.

### Sample size and sampling technique

The sample size was determined using STATA/se version-14 by considering the following statistical assumptions: two sided significance level (α = 5%), Z_a/2_ = Z value at 95% confidence interval = 1.96, power 80% and p = 82% cumulative occurrence of cure rate, 1.78 HR [13]. Accordingly, a total of 440 SAM children’s records were recruited.

$$e=\frac{(\frac{Za}{2}+ZB)2}{\theta^{2}p.(1-p)}\Rightarrow n=\frac{e}{p(e)}$$

θ=lnHR

$$HR=e^{\theta }$$

Where e = event                            p = cumulative occurrence of cure rate

        Z_a/2_ = Z value at 95% confidence interval = 1.96                    HR = hazard ratio

        N = sample size                                                                                        Z_B_ = power of the study

A simple random sampling technique was used to take a random sample from the sampling frame (medical registration number). Open-Epi software version-3 was used to generate random numbers. First serial number or a unique SAM number was extracted from patient registration, and entered from small to the highest into software to select a sample of 440 complete records.

### Data collection procedure

A checklist was adapted from the standard treatment protocol for the management of SAM, monitoring multi chart, registration log book and reviewing relevant literature to extract the required information (S1 Table). Data extracting checklist was revised by using standardized entry based on a regular data registration protocol. Three professional data collectors and one supervisor were recruited, who are trained and experienced in SAM management. Moreover, additional two days training was provided for data collectors to update on the data collection process.

Cured defined as those children who have become free from medical complications, edema and have achieved and maintained sufficient weight gain (when they reach 85% weight for length) [24]. Cure rate is computed as a number of SAM children discharged after cured divided by the total number of SAM children admitted at inpatient TFU [24]. SAM defined as weight for height below -3 z scores of the median WHO growth standards or presence of bilateral edema or mid upper arm circumference < 115mm for a child ≥6months age. Comorbidities was considered as children with SAM, who have TB, and/or HIV and/or malaria and/or severe anemia co-infection at admission to stabilization center [25].

### Data processing and analysis

Data were entered, edited and cleaned by Epi-Info version-7 and analysis was carried out using STATA/se version-14. Exploratory data analysis was undertaken to describe and check outlier, missing and multi-coliniarity variables. Cox proportional hazard model was carried out to estimate time to cure and identify related factors. Hazard Ratio (HR), 95% CI and p-value was used to determine the strength of association and statistical significance. Variables significant at a p-value < 0.25 in the bivariable analyses were eligible to enter into the final multivariable analysis to identify predictors of time to cure. Final statistical test was declared significant at P <0.05.

Cox regression model fitness to the data and proportional hazard assumptions were checked by using both log-log plot and Schoenfeld residuals test.

## Results

### Socio-demographic characteristics

From a total of 416 SAM records, half (50%) children were females and 39.2% were 12–23 months of age (median age = 18 months). More than three-fourth (78.6%) of children came from rural areas (**Table 1**). Regarding special and routine medications, 66.3% and 76.4% children admitted with SAM received vitamin A and folic acid, respectively. Furthermore, the majority of patients (92.8%) took F75 followed by F100 (61%) (**Table 2**).

**Table 1:** Socio-demographic characteristics of SAM children aged 6–59 months admitted in UOGCSH, Northwest Ethiopia, 2017 (N = 416).

Characteristics	Frequency	Percent (%)
**Age(Months)**		
6–11	66	15.9
12–23	163	39.2
24–35	106	25.5
>36	81	19.5
**Sex**		
Male	288	50.0
Female	288	50.0
**Residence**		
Urban	89	21.4
Rural	327	78.6

**Table 2:** Medication provision and mineral supplementation in the therapeutic center of UOGCSH (N = 416).

Variables	Frequency	Percent (%)
**Vitamin A**		
Yes	276	66.4
No	140	33.7
**Folic acid**		
Yes	317	76.4
No	98	23.6
**Deworming**		
Yes	56	13.5
No	358	86.5
**Anti-malaria**		
Yes	20	4.8
No	396	95.2
**Antibiotics**		
Yes	228	54.8
No	188	45.2
**Resomal**		
Yes	263	63.2
No	153	36.8
**IV-fluid**		
Yes	92	22.1
No	324	77.9
**IV-antibiotics**		
Yes	303	72.8
No	113	27.2
**Blood transfusion**		
Yes	49	11.8
No	367	88.2
**Intake of F75**		
Yes	386	92.8
No	30	7.2
**Intake of F100**		
Yes	254	61.1
No	162	38.9

IV-fluid = Intravenous fluid, IV-antibiotics = intravenous antibiotics

## Treatment outcomes

**Fig 1:**
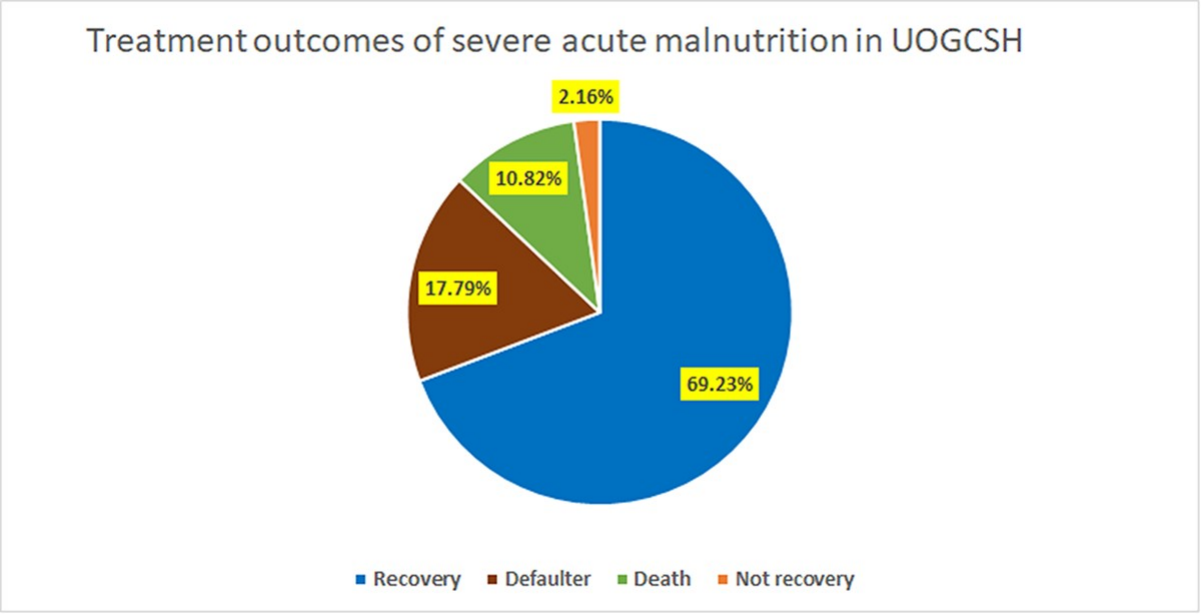
Treatment cure rate of 6–59 months old children with SAM.

**Fig 2:**
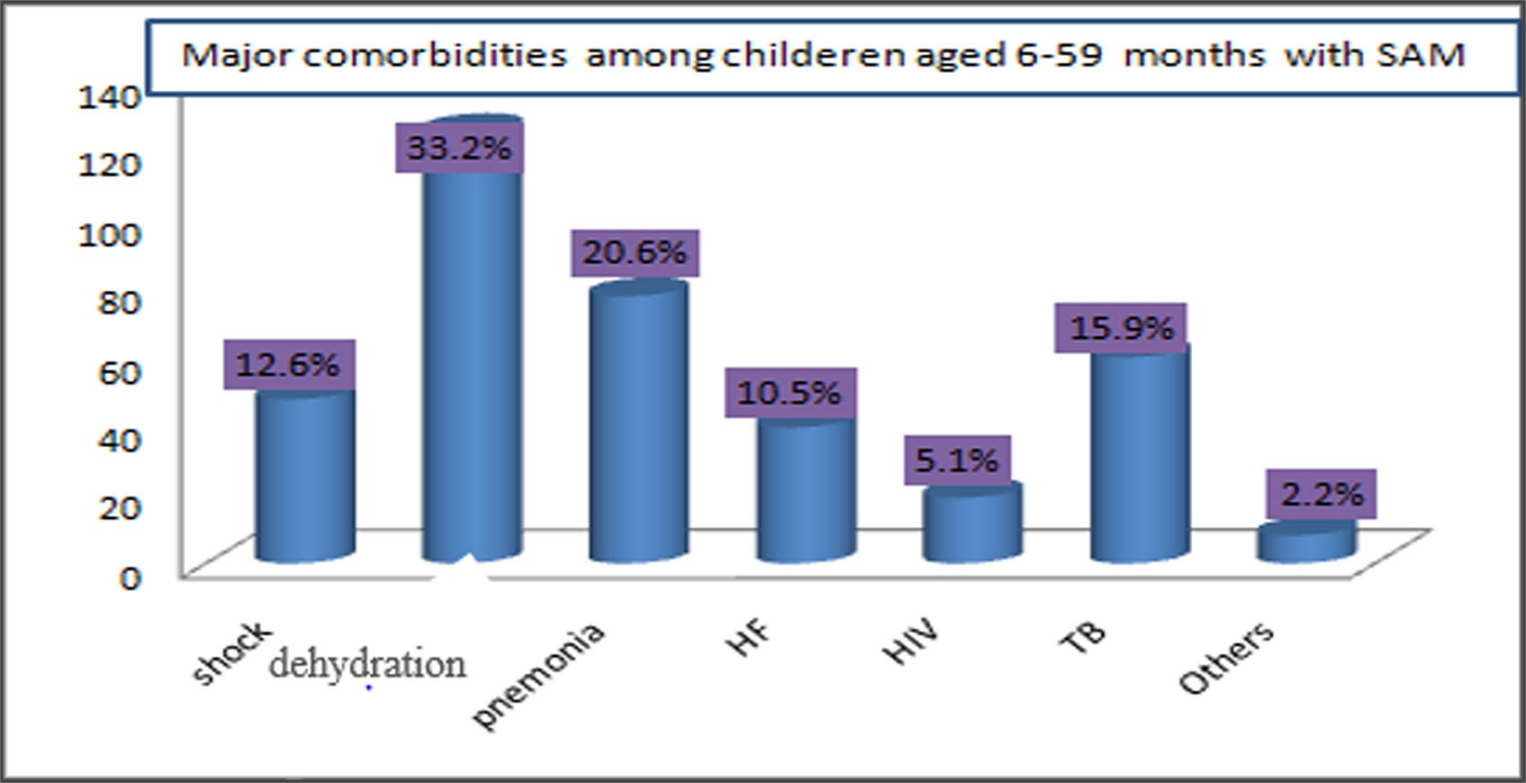
Major medical comorbidities among 6–59 months old children with SAM.

Regarding treatment outcomes of SAM, 288 (69.2%) children were cured while 45(10.8%) died (**Fig 1**). Among admitted children, the most frequent co-morbidities were dehydration (33.2%), pneumonia (20.6%) and tuberculosis (15.9%) (**Fig 2**).

Likewise, marasmus was the predominant (61.2%) types of malnutrition. More than three-quarters (76.6%) of kwashiorkor diagnosed children recovered, with 8.4% death and 14% defaulter rates (**Table 3**).

**Table 3:** Treatment outcomes by types of SAM diagnoses in the TFC of UOGCSH, 2017 (N = 416).

**Types of SAM**	**Treatment outcomes**		
	Cured	Defaulter	Death	Not-cure	Total
**Marasmic**	173(67.8%)	54(21.2%)	22(8.6%)	%)	255(100%)
**Kwashiorkor**	82(76.6%)	15(14.0%)	9(8.4%)	1(0.9%)	107(100%)
**Marasmic-kwashiorkor**	33(61.0%)	5(9.3%)	14(26%)	2(3.7%)	54 (100%)
**Total**	288(69.2%)	74(17.8)	45(10.8)	9(2.2)	416 (100%)

### Survival estimates for time to cure

**Fig 3:**
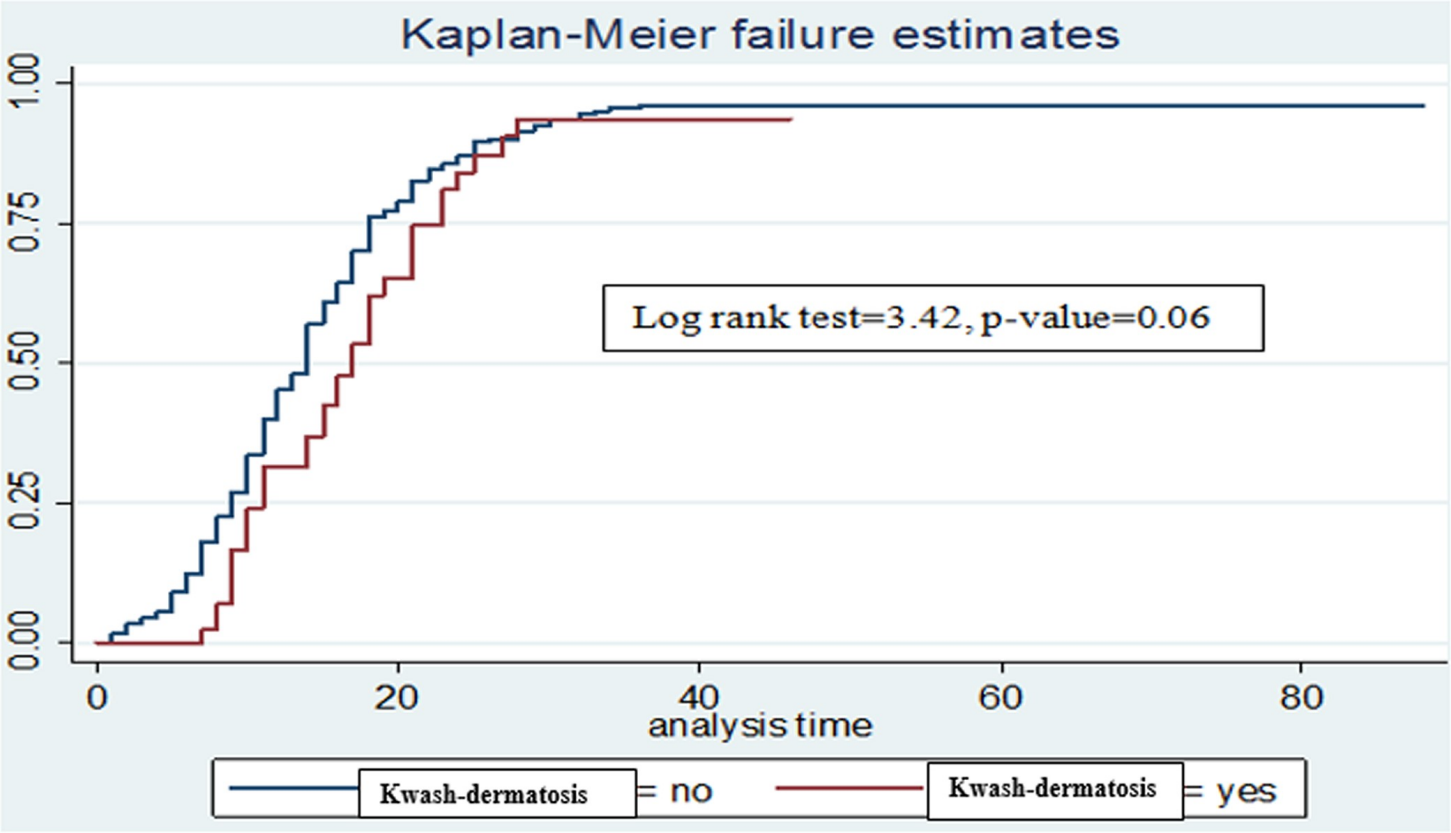
Log rank survival estimates for time to cure among SAM children with kwash-dermatosis.

**Fig 4:**
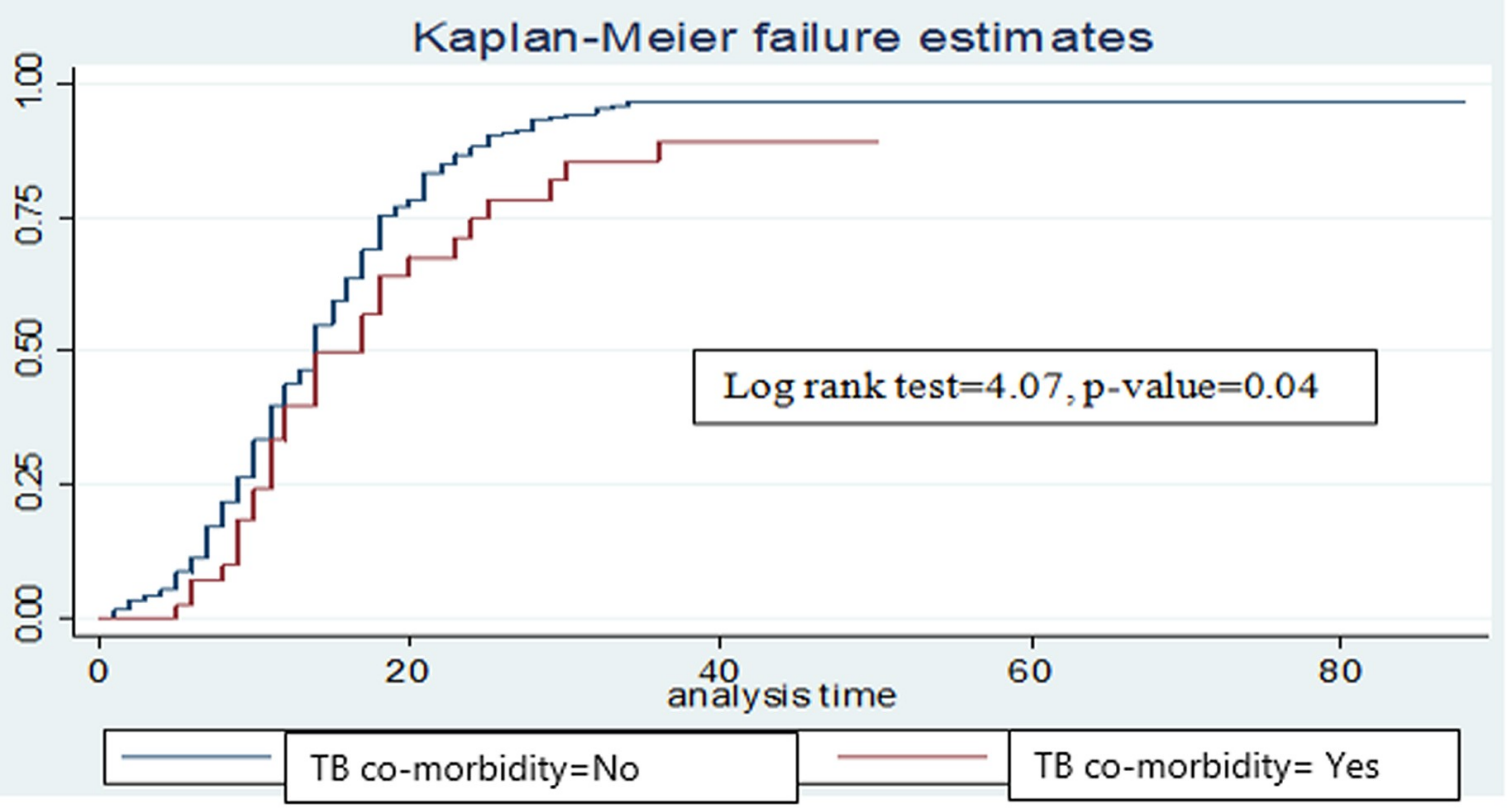
Log rank survival estimates for time to cure among SAM children with TB disease.

**Fig 5:**
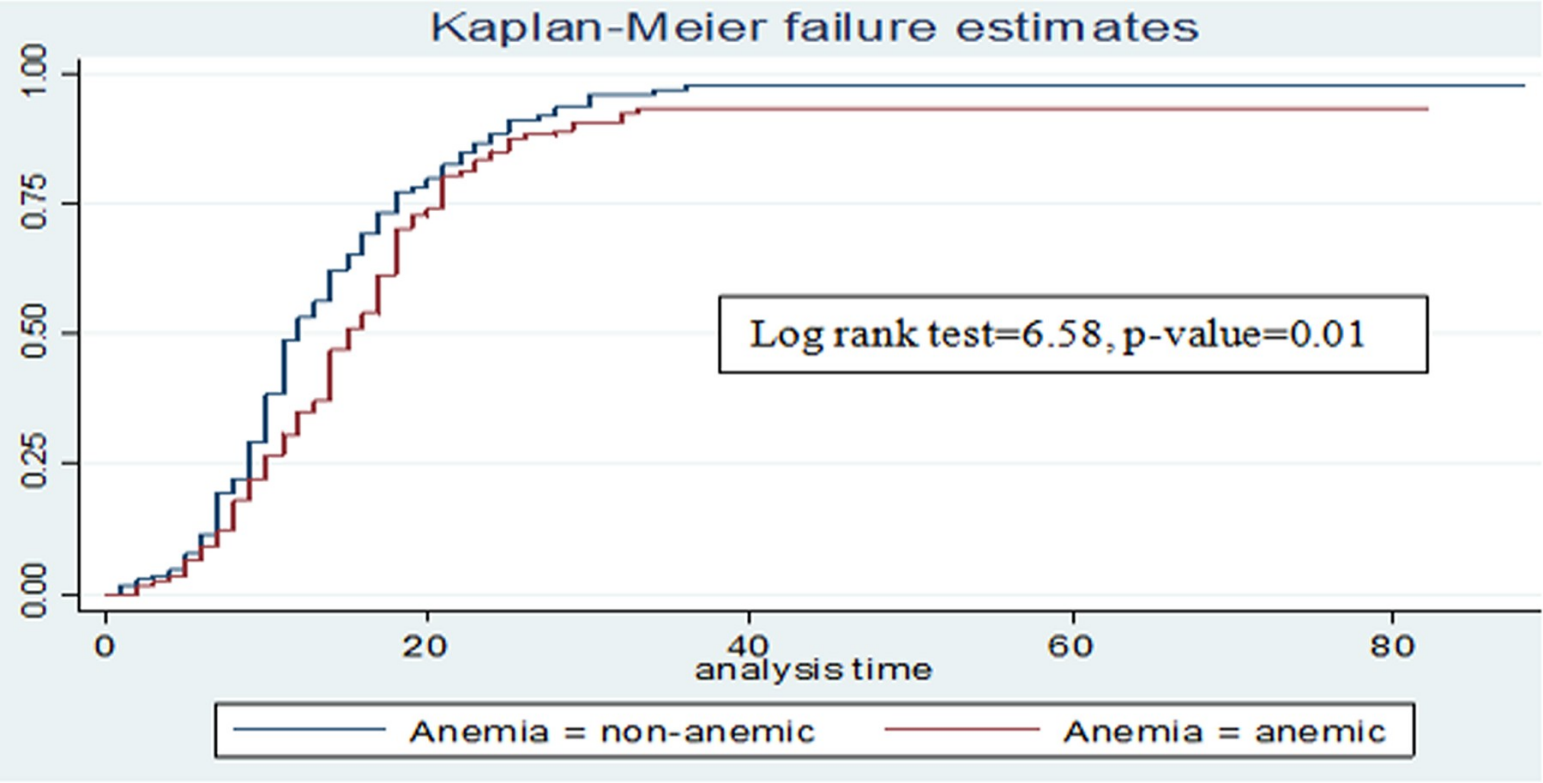
Log rank survival estimates for time to cure among SAM children with anemia.

The median cure time was 11 days (95% CI: 13, 15). There was no difference in the cure time between SAM children with kwash-dermatosis and those without kwash-dermatosis (p-value>0.05) in the log rank survival curves (**Fig 3**). The average length of stay in the hospitals was 18 days (**Table 4**). However, children with anemia or tuberculosis stayed longer before cure than those SAM children without anemia or tuberculosis (p-value < 0.05) (**Figs 4 and 5**).

**Table 4:** Performance indicator values of inpatient therapeutic feeding centers at UOGCSH as compared to the SPHERE standard guideline.

Performance indicator	UOGCSH	Sphere project reference value	
		Acceptable	Alarming
**Cure rate**	69.2%	>75%	<50%
**Death rate**	10.8%	<10%	>15%
**Defaulter rate**	17.8%	<17%	>25%
**Length of stay**	18 days	<28days	>42days

### Predictors related with time to cure

In bivariable Cox regression analyses, kwash-dermatosis, anemia, altered temperature and shock were significantly associated with time to cure in SAM children. However, after controlling for potential confounders (in multivariable Cox regression analysis), comorbidities at admission including kwash-dermatosis, anemia, tuberculosis and altered body temperature at admission were significantly associated with time to cure in SAM children on treatment. Children without kwash-dermatosis had 1.48 times higher cure rate compared to children with kwash-dermatosis (AHR: 1.48 (95% CI: 1.01, 2.16)). Similarly, SAM children with no anemia had 1.36 times higher probability of cure than anemic children (AHR: 1.36(95% CI: 1.07, 1.74)). In addition, children without tuberculosis had about 1.6 times higher probability of cure as compared to their counterparts (AHR: 1.6(95% CI: 1.04, 2.43)). Finally, children with normal body temperature at admission had 1.58 times higher probability of cure in comparison with children with altered body temperature (AHR: 1.58(95% CI: 1.04, 2.4)) (**Table 5**).

**Table 5:** Bivariable and multivariable Cox-regression analysis of factors associated with time to cure among children with SAM at UOGCSH, 2017.

Variables	Event	Censored	CHR	AHR
**Age**				
6–23 months	179	50	1.3(0.81–1.41)	1.19(0.94–1.29)
24–59 months	109	78	1	1
**Sex**				
Male	157	51	1.39(1.1–1.45)	1.2(0.77–1.23)
Female	131	77	1	1
**Kwash-dermatosis**				
Yes	34	19	1	1
No	254	109	1.37(1.1–1.97)	1.48(1.01–2.16) **
**Pneumonia**				
Yes	45	12	1	1
No	243	116	0.89(0.63–1.2)	0.93(0.66–1.3)
**Heart failure**				
Yes	17	12	1	1
No	269	115	1.24(0.76–2)	1.48(0.89–2.46)
**Deworming**				
Yes	41	15	0.89(0.6–1.2)	0.8(0.56–1.14)
No	247	111	1	1
**Folic acid**				
Yes	229	88	1.1(0.82–1.47)	1.18(0.86–1.62)
No	58	40	1	1
**Pulse**				
Normal	160	41	1	1
Altered	128	82	0.8(0.63–1.02)	0.82(0.64–1.05)
**Anemia**				
Anemic	143	84	1	1
Non-anemic	132	43	1.34(1.1–1.7)	1.36(1.07–1.74) **
**TB disease**				
Yes	28	16	1	1
No	260	112	1.47(0.99–2.17)	1.6(1.04–2.43) **
**HIV test**				
Reactive	9	5	0.74(0.37–1.46)	0.82(0.41–1.64)
Non-reactive	170	91	0.8(0.6–1.03)	0.83(0.64–1.08)
Un-known	108	32	1	1
**Temperature at admission**				
Normal	260	103	1.56(1.08–2.3)	1.58(1.04–2.4) **
Altered	28	25	1	1
**Shock**				
Yes	6	30	1	1
No	282	96	2.63(1.08–6.38)	1.98(0.80–4.9)

** Significant predictors in the multivariable analysis at P<0.05.

HIV = Human Immune-Deficiency Virus, TB = Tuberculosis

## Discussion

The current study determined treatment cure rate and its predictors among 6–59 months old children with SAM admitted to hospital’s stabilization center. The study found a cure rate of 69.2% which was unacceptably low when compared to the sphere standards that recommend the cure rate should exceed 75% [26] in malnourished children on relevant treatment protocol. This low cured rate may be attributable to a late presentation [27], higher defaulter rate and patient overload [15]. As well, this low cure rate may be attributable to non-adhering with the standard protocol for management of SAM [15, 28]. Thus, to achieve a better cure rate, the management of SAM standard protocol needs to be implemented properly. This means that strengthening outpatient treatment programme should tackle barriers to access, encourage early identification of SAM, reduce inpatient caseloads and decrease the risks of cross-infection [29, 30]. Lastly, in this study achieving low cure rate may be because of mismanagement of children such as partial prescription of routine medication and due to comorbidity at admission like a presence of pneumonia and tuberculosis. However, the average length of hospital stay (i.e., 18 days) is less than the sphere international standard set length of hospital stay (i.e., <28 days). The median cure time was consistent with other studies done in Karat and Fasha, and Debre Markos and Finote Selam stabilization centers [10, 13], although the findings showed a wide range of variations in the cure rate as compared to other studies in other parts of the country [10, 11, 13, 15–17, 31, 32]. This could be due to differences in socioeconomic status, quality of health care provision, availability of therapeutic feeding and special medications[33].

The current study also found high mortality rate than those reported in regions of Ethiopia [10, 13, 18] and Malawi [32]. The possible explanation for these discrepancies in SAM mortality rate could be due to the differences in the causes of SAM in various parts of the world [34]. Also, that variations in mortality may be associated with the hospital health care quality, variability in the socioeconomic status of catchment populations to the staffing ratios, caseload and many more [35]. The other possible explanation for this variation might be due to delay in seeking care results in medical complication at the time of hospitalization for SAM or late arrival at hospital that may explain the associated high mortality rate observed [27].

The prognosis of SAM largely depends on the presence of other comorbidities at admission. The possible reason might be that these children depressed humeral and cell-mediated immunity are attributable reasons for the prevalence of infection. For instance, children without kwash-dermatosis had higher probability of cure compared to children kwash-dermatosis. The possible reason might be the fact that children with kwash-dermatitis were prone to develop infection and metabolic complications, and found to be edematous with more skin lesion which in turn lead to more complications and would take longer time to cure.

Children who were not anemic had higher probability of cure than those children who were anemic, which is in line with the finding from Woldiya [36] and Bahirdar [28]. This is due to the fact that there is an increase in the prevalence of infection and increased probability of heart failure in anemic children leading to prolonged time to cure [37]. Likewise, SAM children without TB disease were more likely to cure earlier than those with TB. Consistent with this finding, studies from Bahirdar referral hospital and Jimma university specialized hospital TFCs revealed that less recovery and a more likely risk of death in children with co-morbidities such as Tb disease [15, 18]. This might indicate that a child with co-morbidities requires a prolonged hospital stay, present with an increased nutritional crisis, and more nutrient requirement because of reduced appetite and nutrient absorption in comparison with their counterparts [38].

Furthermore, altered body temperature at admission was also another important predictor of cure rate of SAM children admitted to TFC. That is, children with normal body temperature at admission had increase probability of cure by 58% as compared to those children with altered body temperature. This finding is consistent with other studies done in Gedeo zone [17] and Dilla referral hospital [19]. Since being critical at admission, hypothermia and hyperthermia affect biochemical reaction of the body; and they are indicators of altered metabolism and serious infections that attributed to reducing recovery [24]. Generally, SAM children with comorbidities require prolonged hospital stay and increased nutritional requirement because of reduced appetite and nutrient absorption in comparison with their counterparts [38].

As a study limitation, despite these interesting findings with policy and practical implications, the use of data collected from secondary sources and subsequent incompleteness might affect the reliability of the findings. Relevant variables like family educational status, income, socioeconomic status and maternal nutritional status were inadequately recorded and were not included in the analysis.

## Conclusion

The cure rate in SAM children was low relative to sphere standard guideline. Prognosis for SAM treatment largely depends on the presence of other comorbidities at admission. Available intervention modalities need to address coexisting morbidities to achieve a better cure rate in SAM children.

## Supporting information

S1 Table Abstraction tool P treatment.docx. (DOCX)

## Abbreviations

HIV/AIDS: Human Immune-Virus /Acquired Immune Deficiency Syndrome
MUAC: Mid-Upper-Arm-Circumference
NGT: Naso-Gastric Tube
NGT: Naso-Gastric Tube
SAM: Severe Acute Malnutrition
TB: Tuberculosis
TFC: Therapeutic Feeding Center
UOGCSH: University of Gondar Comprehensive Specialized Hospital
WFH: Weight for Height
WHO: World Health Organization
