# Supplementary material for: Treatment cure rate and its predictors among children with severe acute malnutrition in northwest Ethiopia: A retrospective record review
Source: PLoS One. 2019 Feb 20;14(2):e0211628. doi: 10.1371/journal.pone.0211628 (PMC6382114; doi:10.1371/journal.pone.0211628)
Supplement: S1 Table — (DOCX) [file pone.0211628.s001.docx]

**Annex 1. Data extraction format University of Gondar College of medicine and health sciences school of nursing department of pediatrics nursing of post graduate studies research on treatment outcome among Children with Severe Acute Malnutrition Admitted to Stabilization Center.**

**INFORMED CONSENT**

This data extraction format is intended to assess predictors of mortality under five children with severe acute malnutrition admitted to therapeutic feeding units of UOGCSH. The study will be conducted through reviewing patient records. The study is aimed to fill the information gap and provide empirical evidence for program planner, decision makers and SAM program implementer at the different level by enabling them to access a baseline data on treatment outcome. Moreover it will be a paramount important to curb the morbidity and mortality due to severe acute malnutrition of the disease. And it assists in the development of a system for improving the child survival program in stabilization center.

Date of review __/___/_____

Name of the reviewer ______________________________ Signature ____________

Time (Started/ Ended) ________/_________

Supervisor Name _______________________________

Signature _______________ Date __/____/_______

Total number of records reviewed ________

Reviewed Patient’s card No. from ________to_______

Available Data: I. Complete____ II. Incomplete_____ III. Excluded____

_________________________________________________________________________________________________________________________________________________________________________________________________________________________________________________________

***(Please use additional blank paper if the space is not enough)***

**Part I. patient baseline Information (filled from therapeutic registration book)**

| **SECTION 1. DEMOGRAPHIC CHARACTERISTICS** | | | | | | |
| --- | --- | --- | --- | --- | --- | --- |
| ***No*** | | **VARIABLES** | **CODING CATEGORIES** | | **Remark** | |
| ***101*** | | Patient ID Number | _______________ | |  | |
| ***102*** | | Unique SAM Number | _______________ | |  | |
| ***103*** | | Age | _______(month) | |  | |
| ***104*** | | Sex | 1. male  2. female | |  | |
| ***105*** | | Residence | 1. urban  2. rural | |  | |
| ***106*** | | New admission | 1. Yes  2. No | |  | |
| ***107*** | | Admission date | -----------dd/----------mm-/----------yyy | |  | |
| ***108*** | | Admission time | Time-----------Am/Pm | |  | |
| ***109*** | | Readmission (check former ID) | 1. Yes  2. No | |  | |
| ***110*** | | Admission date | _______dd/-------------mm/-------------yyy | |  | |
| ***111*** | | Admission time | Time --------Am/Pm | |  | |
| **Section 2. Anthropometric characteristics at admission** | | | | | | |
| ***201*** | Height | | | ------------(cm) | |  |
| ***202*** | Weight | | | -------------(kg) | |  |
| ***203*** | Wt/Ht | | | ---------------(%) | |  |
| ***204*** | MUAC | | | -------------(cm) | |  |
| ***205*** | Appetite at admission | | | 1. Failed appetite  2. Passed appetite | |  |
| ***206*** | Has history of Bottle feeding? | | | 1. yes  2. no | |  |
| ***207*** | Oedema | | | ( 0 , + , ++ , +++ ) | |  |

NB (0) no edema,

(+): grade one edema

(++): grade two edema (moderate) and

(+++): grade three edema (generalized edema).

| **SECTION 3. severe acute malnutrition diagnosis** | | | | | |
| --- | --- | --- | --- | --- | --- |
| **NO** | | **VARIABLES** | | **CODING CATEGORIES** | **Remark** |
| 301 | | Severe acute malnutrition type | | 1. Marasmus  2. Kwashiorkor  3. Marasmic-kwashiorkor |  |
| 302 | | Major co-morbidities | | 1. TB  2. HIV  3. Malaria  4. Diarrhea  5. Pneumonia  6. Heart failure  7. Other(specify)________ |  |
| 303 | | Complication | | 1. Dehydration  2. Shock  3. Skin lesion  4. Pale-conjunctiva  4. Others(specify)_____ |  |
| **Lab test results** | | | | | |
| **401** | Hgb | | -----------g/dl | |  |
| **402** | Malaria blood film | | 1. positive  2. Negative | |  |
| **403** | TB | | 1. Yes  2. No | |  |
| **404** | HIV test | | 1. Reactive  2. Non-reactive | |  |

| **SECTION 4. management of severe acute malnutrition** | | | | |
| --- | --- | --- | --- | --- |
| NO | **VARIABLES** | | **CODING CATEGORIES** | **Remark** |
| **Therapeutic diet** | | |  |  |

| 501 | | | Phases | | | 1. Phase 1  2. Transition Phase  3. Completed both phase | |  |
| --- | --- | --- | --- | --- | --- | --- | --- | --- |
| 502 | | | Diet name | | | 1. F -75  2. F-100 | |  |
| 503 | | | Milliliter/feed | | | 1. F-75 ---------ml/feed  2. F-100 --------- ml/feed | |  |
| 504 | | | Number of feed per day | | | 1. F-75 ---------feed/day  2. F 100 ----------feed/day | |  |
| 505 | | | Additional food | | | 1. Porridge  2. Family meal | |  |
| 506 | | | Feed by | | | 1. Orally  2. By naso-gastric tube( NG –tube) | |  |
| **Section 5 Routine Medications** | | | | | | | | |
| 601 | | Vitamin A | | | 1. yes  2. no | | |  |
| 602 | | Folic acid | | | 1. yes  2. no | | |  |
| 603 | | Anti-malaria treatment | | | 1. yes  2. no | | |  |
| 604 | | De-worming | | | 1. yes  2. no | | |  |
| 605 | | Antibiotic | | | 1. Amoxicillin  2. Other _____________ | | |  |
| **Special medications** | | | | | | | | |
| 701 | ReSomal | | | 1. yes  2. no | | |  | |
| 702 | IV fluids | | | 1. yes  2. no | | |  | |
| 703 | IV antibiotics | | | 1. yes  2. no | | |  | |
| 704 | Blood transfusion | | | 1. yes  2. no | | |  | |

| **Section 6 Discharge and outcome** | | |  |
| --- | --- | --- | --- |
| 801 | Discharged as | 1. recovered  2. defaulted  3. died  4. non-recovered |  |
| 802 | Discharge weight | **_________kg** | |
| 803 | Length of stay | __________days | |
| 804 | Discharged date | -------------dd/--------mm/----------yyy | |
| 805 | Discharged time | -----------Am/Pm | |
